# Arthroscopic Anterior Cruciate Ligament Reconstruction Using Iliotibial Band Autograft With Bone Block

**DOI:** 10.1002/atn2.70393

**Published:** 2026-11-13

**Authors:** Danlei Huang, Weizong Weng, Jun Wang, Zhiyang Ye, Guangze Wang, Xiao Chen, Jianming Huang

**Affiliations:** ^1^ Department of Orthopedics The 73th Group Military Hospital of People's Liberation Army Xiamen Fujian China

## Abstract

Anterior cruciate ligament (ACL) reconstruction is the first‐line treatment for ACL injury, whereas existing autologous grafts each have inherent limitations. This study describes the use of an iliotibial band (ITB) graft with a bone block for ACL reconstruction, designed as a femoral bone block combined with tibial tendon configuration, which precisely matches the anatomical attachment and biomechanical loading characteristics of the native ACL. The surgery is performed through arthroscopic evaluation, harvesting and weaving of the ITB with a bone block, creation of femoral and tibial bone tunnels, and graft introduction and fixation. This graft features the advantage of easy harvesting of the ITB, whereas the femoral bone column allows for rapid bone‐to‐bone healing, enhances stability within the femoral tunnel, and reduces the risk of tunnel widening and femoral‐sided rerupture. Tibial‐sided tendon fixation avoids excessive tibial tunnel enlargement and simplifies surgical steps. Arthroscopic ACL reconstruction using the ITB with bone block is technically straightforward and provides high initial stability. It offers another graft option for both primary treatment of ACL injury and revision surgery after failed reconstructions and is particularly suitable for patients with multiple previous tendon harvests from the same knee or those requiring large‐diameter grafts because of bone tunnel enlargement.

## Graphical abstract

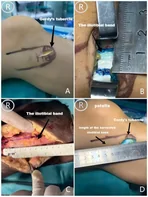

**VIDEO 1:** Palpate and mark Gerdy's tubercle on the knee skin. An oblique incision of approximately 2 cm is made along the course of the iliotibial band (ITB). A 1‐cm‐wide harvesting area of the ITB is delineated with methylene blue. A ruler is used to measure a 10‐cm graft length for skin marking. The ITB graft is harvested under arthroscopic supervision and prepared for use. Arthroscopic exploration is performed to assess the injury of the anterior cruciate ligament (ACL) of the knee joint. The anteromedial femoral insertion of the ACL is exposed. The femoral ACL footprint is localized using a Femoral Introfix guide, and a Kirschner wire is drilled through. An 8‐mm cannulated drill is used to create a 1.5‐cm medial femoral tunnel, which is further overdrilled with a 4.5‐mm cannulated drill and prepared. The integrity of the inner wall of the femoral tunnel is confirmed. The total length of the femoral tunnel is measured. A suture passer is introduced through a pin with a hole to place a guiding suture. A tibial guide is positioned adjacent to the remnant ACL tibial insertion (at the intersection of the anterior horn of the meniscus and the perpendicular line to the tibial intercondylar eminence). A Kirschner wire is drilled through, followed by overdrilling with a 7‐mm cannulated drill and preparation, and the guiding suture is brought out. The prepared ITB with a bone plug is introduced into the joint starting from the femoral side, ensuring that the miniplate passes through the tunnel for suspension fixation. Traction is continued on the pulling suture until the bone block is fully seated within the bone tunnel, and the other end of the graft is pulled out through the tibial tunnel. No impingement of the graft within the joint is confirmed. Video content can be viewed at https://doi.org/10.1002/atn2.70393.

Anterior cruciate ligament (ACL) reconstruction (ACLR) is the first‐line treatment for ACL injury, aiming to restore knee stability, facilitate return to sports, and delay the progression of knee arthritis.^1^, ^2^ Currently, no consensus exists regarding the optimal graft in clinical practice, although autologous tendons are most widely used. Bone–patellar tendon–bone (BPTB) graft exhibits excellent osseous healing and favorable biomechanical properties, yet it carries donor‐site complications including anterior knee pain. Modified quadriceps tendon grafts with rectangular bone blocks have gained increasing attention; compared with conventional BPTB, they provide stronger tendon‐bone integration and reduce the risk of patellar tendon rupture.^3^, ^4^ Moreover, the bone block is situated at the femoral aspect. From a biomechanical standpoint, the tensile force of the ACL is predominantly concentrated at its femoral insertion during knee flexion‐extension and rotation;^5^ rigid fixation of the bone block can directly resist this load and prevent graft elongation or slippage under high tension. However, this technique requires specialized instruments and has a relatively long learning curve. Accordingly, integrating the advantages of current mainstream grafts while overcoming their limitations, our team uses an iliotibial band (ITB) with a bone block as a graft for ACLR. This graft allows rapid bone‐to‐bone healing in the femoral tunnel, with sufficient mechanical strength and healing potential to meet the demands of ACLR. The “femoral bone block + tibial tendon” design closely mimics the native ACL insertion anatomy. It offers another graft option for patients with primary or recurrent ACLR failure, especially those with multiple ipsilateral tendon harvests and tunnel enlargement requiring large‐diameter grafts (Table 1).

**TABLE 1:** Indications and Contraindications

Indications
• Primary ACL injury in noncompetitive athletes.
• Complicated with patellar tendinopathy, hamstring, or ankle dysfunction.
• ACL revision surgery for tunnel widening or graft loosening after primary reconstruction.
• Patients with poor bone healing capacity.
• Patients with high requirements for postoperative rehabilitation cycle.

Contraindications
• Pathologies at iliotibial band donor site or iliac bone pillar harvest site.
• Severe knee osteoarthritis.
• Combined severe multiple knee ligament injuries.
• MRI showed lateral complex injury or positive pivot‐shift test.

ACL, anterior cruciate ligament; MRI, magnetic resonance imaging.

## SURGICAL TECHNIQUE

### Patient Position, Portal Creation, and Arthroscopic Examination

The patient is placed in the supine position (Ethics Approval Number: 73JYY2026215483). The affected limb is routinely disinfected, and a tourniquet is applied. Anterolateral and anteromedial portals are established as the inflow and working portals, respectively. Sequential arthroscopic evaluation is performed for the articular cartilage, anterior and posterior cruciate ligaments, menisci, and intercondylar notch stenosis. The rupture of the ACL is confirmed intraoperatively. Concomitant meniscal injuries, if present, are repaired (Table 2).

**TABLE 2:** Step‐by‐Step Surgical Procedure

1. Harvest a portion of the iliotibial band and an intact bone block at Gerdy's tubercle (size selected based on specific conditions).
2. Weave the tendinous part of the iliotibial band with high‐strength sutures.
3. Drill a hole in the center of the graft bone block, pass the high‐strength sutures through the bone block, and fix a miniplate.
4. Establish a femoral bone tunnel matching the size of the bone block.
5. Establish a tibial bone tunnel.
6. Introduce the graft into the joint cavity, with both ends passing through the bone tunnels, respectively; ensure that the miniplate flips smoothly outside the femoral tunnel and the bone block is firmly embedded in the femoral tunnel.
7. After pretensioning the tendon, fix the tibial end with an interference screw.
8. Fix the traction suture at the distal end of the tibial segment with an outer‐row suture anchor.

### Harvesting and Weaving of ITB With Bone Block

Gerdy's tubercle and course of the ITB are marked on the body surface. An oblique incision of approximately 2 cm in length is made at Gerdy's tubercle of the tibia. The skin, subcutaneous tissue, and deep fascia are incised to expose the tibial Gerdy's tubercle and the adjacent portion of the ITB. The width of the intended ITB graft (about 1 cm) is marked with methylene blue. Starting from the tendon‐bone junction (where the ITB attaches to Gerdy's tubercle), the graft is planned to extend proximally along the ITB for approximately 10 cm (Figure 1). After subcutaneous undermining, a 10 × 1 cm ITB graft, pedicled distally, is harvested. The ITB is woven into a single‐round tendinous structure (8 mm in diameter) using high‐strength sutures (ULTRABRAID; Smith & Nephew, Andover, MA). The woven tail sutures and the ITB are passed through a cartilage transplantation kit (COR; DePuy Mitek, Raynham, MA). A bone block with a diameter of 10 mm and length of 2 cm is harvested at Gerdy's tubercle (the diameter of the bone block is adjusted according to the patient's body habitus and activity level). The surrounding soft tissues are dissected with tissue scissors to obtain an intact bone block. A 2‐mm Kirschner wire is drilled through the center of the bone block, through which high‐strength sutures are passed to weave the femoral end of the ITB. A miniplate (ENDOBUTTON; Smith & Nephew, Andover, MA) is installed on the graft for subsequent fixation (Figure 2).

**FIGURE 1:**
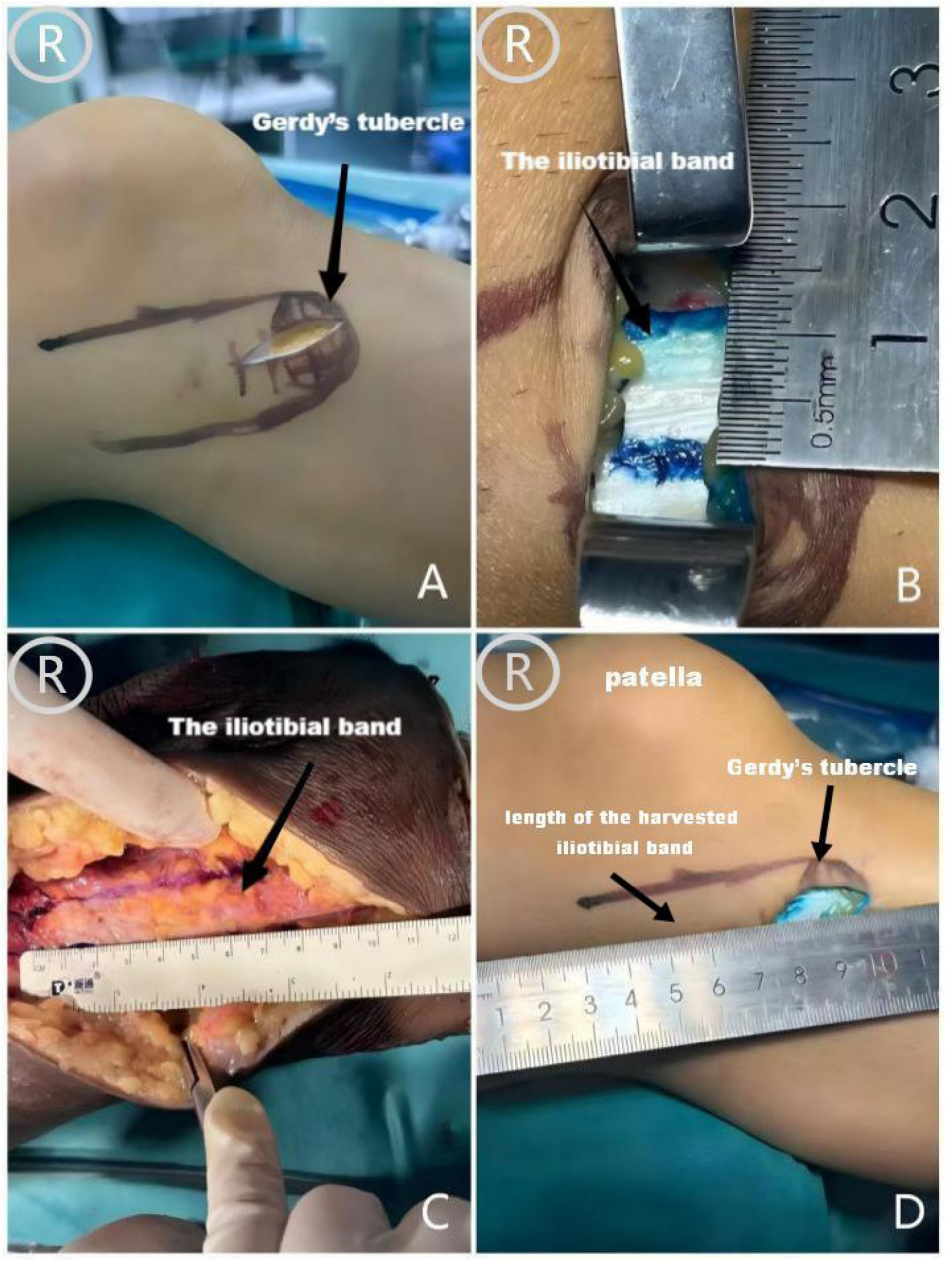
(A) Intraoperative tendon harvest incision at Gerdy's tubercle. (B) Intraoperative width of the harvested iliotibial band (approximately 1 cm). (C) Schematic diagram of the anatomical extent of iliotibial band harvest. (D) Intraoperative length of the harvested iliotibial band (approximately 10 cm). (R, right.)

**FIGURE 2:**
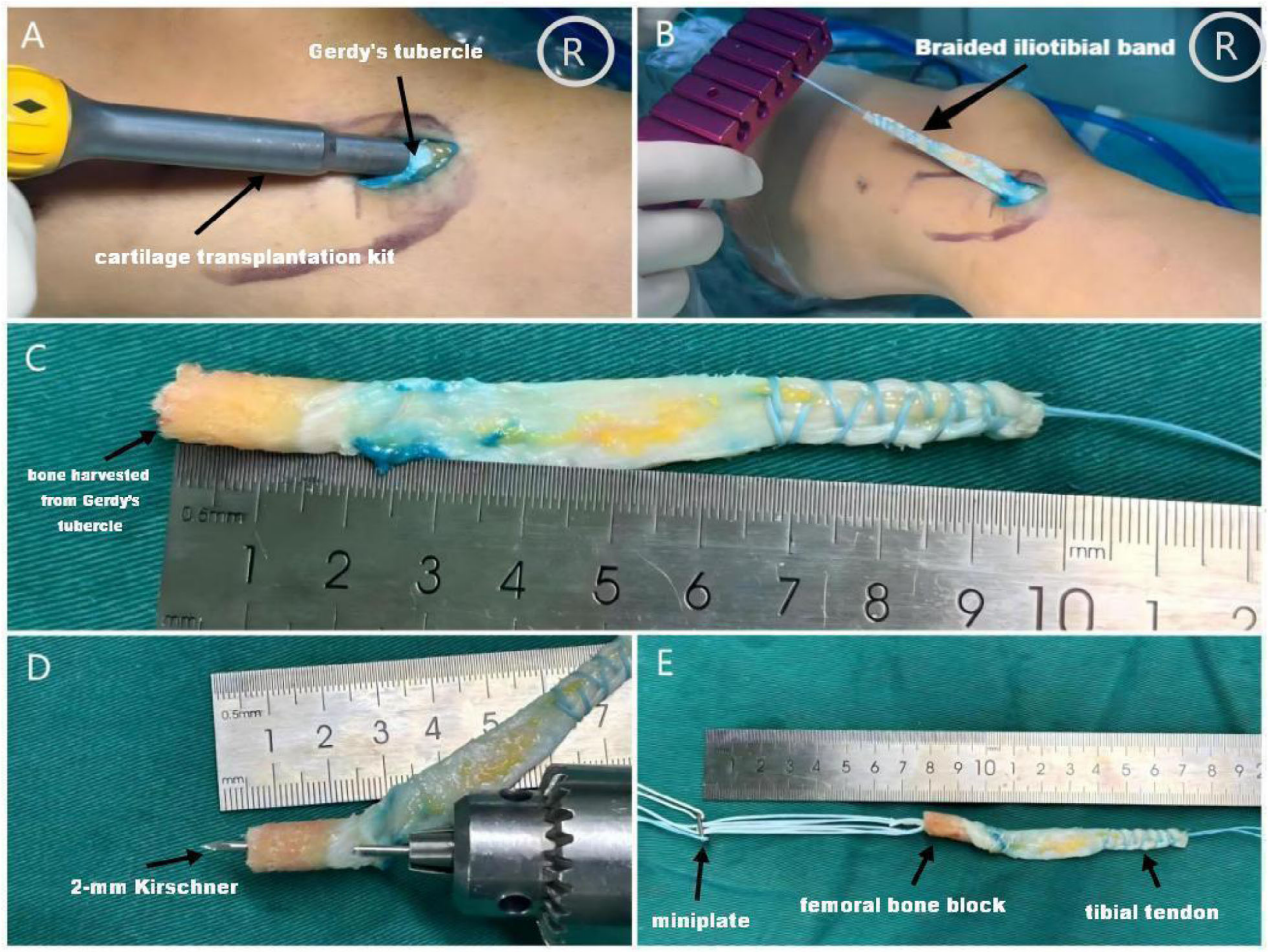
(A) Bone block preparation using the cartilage transplantation kit provided. (B) Weaving of the iliotibial band end with high‐strength sutures. (C) Harvested intact graft. (D) A 2‐mm Kirschner wire drilled through the center of the bone block. (E) Completed graft preparation. (R, right.)

### Femoral and Tibial Tunnel Placement and Creation

The remnant anteromedial attachment of the ACL is exposed, and the femoral intercondylar notch is visualized. A Femoral Introfix guide is inserted via the anteromedial portal to localize the femoral ACL footprint, followed by Kirschner wire drilling. An 8‐mm cannulated drill is used to create a femoral tunnel approximately 1.5 cm medially, and a 4.5‐mm cannulated drill is used for overdrilling and preparation. A tibial guide (Arthrex, Naples, FL) is positioned adjacent to the remnant tibial ACL attachment (at the intersection of the anterior horn of the meniscus and the perpendicular line to the tibial intercondylar eminence). After Kirschner wire drilling, a 8‐mm cannulated drill is used for tibial tunnel preparation (Figure 3).

**FIGURE 3:**
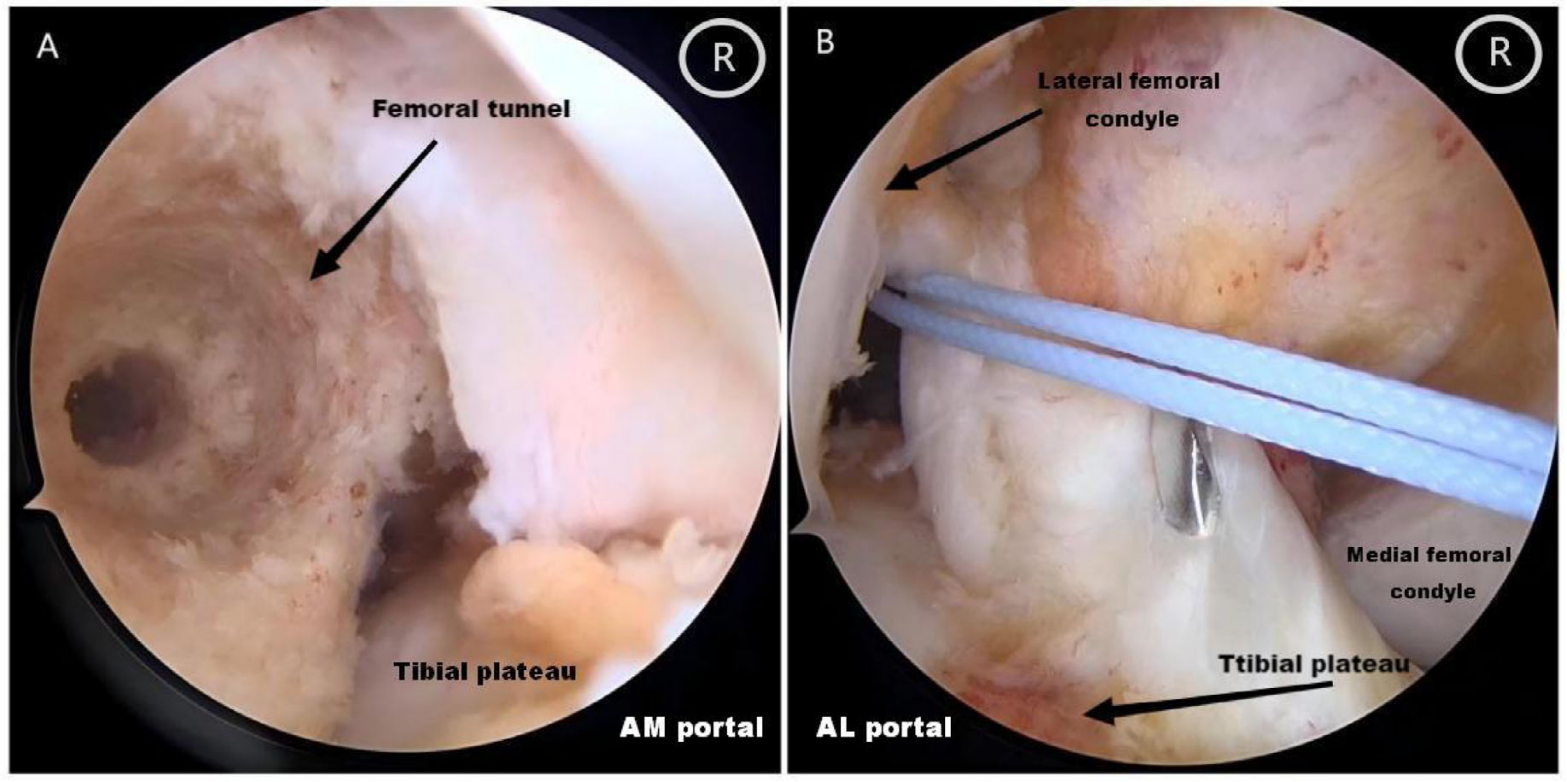
(A) Femoral tunnel preparation. (B) Tibial tunnel preparation. (AL, anterolateral; AM, anteromedial; R, right.)

### Graft Introduction and Fixation

The graft is introduced into the joint cavity through the anteromedial portal and first passed out of the femoral tunnel until the plate flips smoothly outside the tunnel and the bone block is firmly impacted within the femoral tunnel. The other end is then pulled out through the tibial tunnel. The knee is flexed and extended with gentle tension for graft pretensioning. At 30° of knee flexion, an interference screw is used for press‐fit fixation within the tibial tunnel, and the trailing sutures are fixed with a suture anchor. Graft position is confirmed arthroscopically to be satisfactory without impingement (Figure 4). The joint is irrigated, and the incisions are closed. The complete surgical procedure is shown in Video 1. Table 3 includes a list of pearls and pitfalls that the authors find helpful.

**FIGURE 4:**
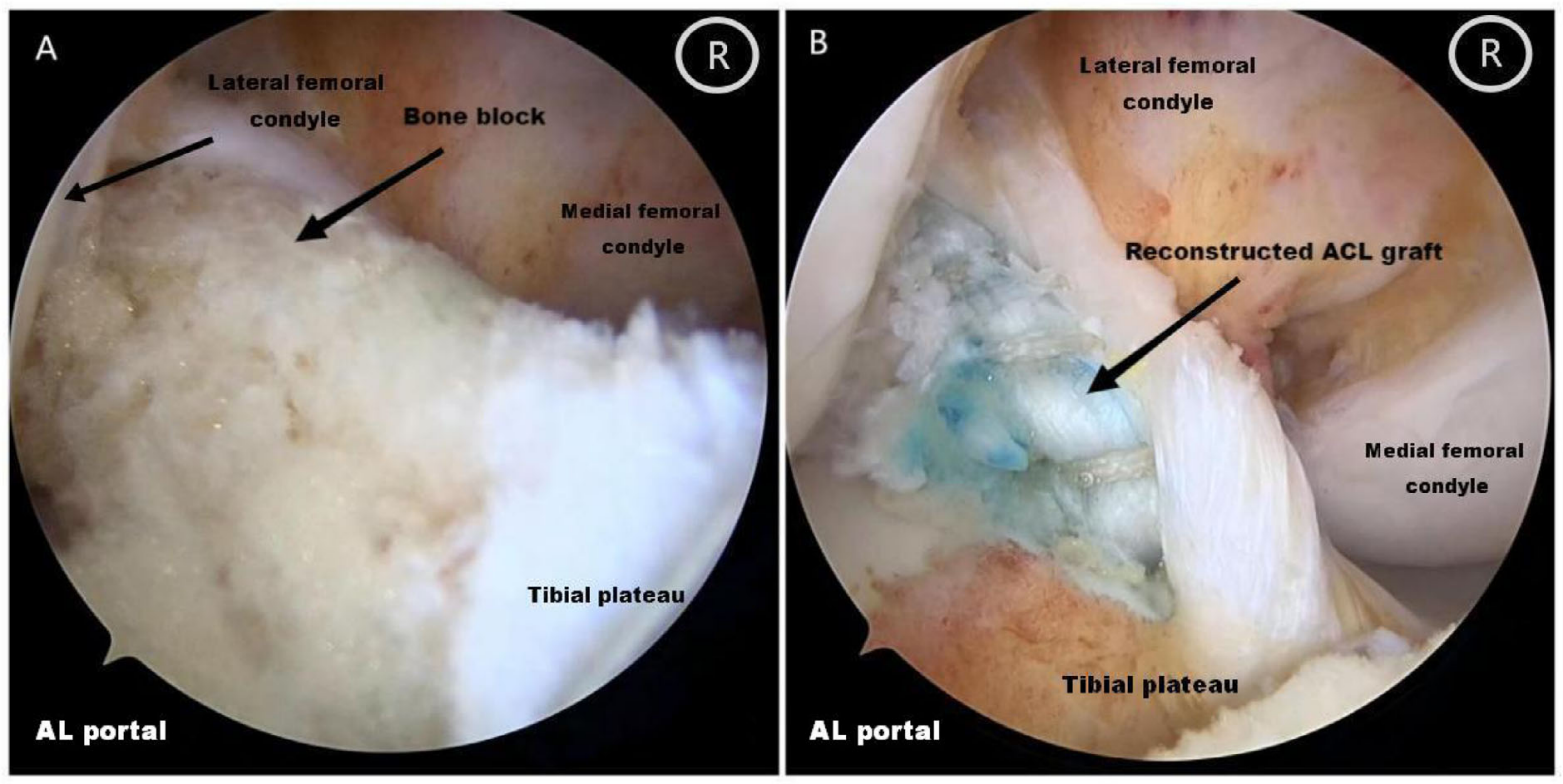
(A) Bone block inserted into the femoral tunnel. (B) Intraoperative confirmation of appropriate graft position. (ACL, anterior cruciate ligament; AL, anterolateral; R, right.)

**TABLE 3:** Pearls and Pitfalls

Pearls
• Graft introduction is recommended to be performed in 2 steps: first, expose the femoral end and ensure the bone block is fully inserted into the bone tunnel with the miniplate smoothly flipped and attached to the femoral cortex; subsequently, pull the graft toward the tibial end through the joint cavity. This approach avoids the risk of bone block impaction or defect at the entrance of the tibial plateau bone tunnel.
• A small lateral longitudinal incision at the knee joint is recommended for graft harvesting, with its center located 2 cm proximal to Gerdy's tubercle. Incision placement should avoid excessive posterior positioning to prevent traction injury to the common peroneal nerve.
• The bone block should only be harvested from the edge of Gerdy's tubercle (diameter ≤ 1 cm), avoiding involvement of the insertion core to prevent injury to the anterolateral complex.
• After bone harvesting, other bone graft substitutes should be implanted at the harvest site to promote healing.

Pitfalls
• The bone block is prone to fracture during insertion into the femoral tunnel, which may preclude its complete seating.
• Gentle handling is required during bone block harvesting and woven suture threading to prevent bone block defects.

## DISCUSSION

Currently, arthroscopic ACLR remains the first‐line clinical treatment for ACL injury, regardless of primary or revision surgery.^6^, ^7^ The first graft used for ACLR was the BPTB, which forms bone‐to‐bone healing at both femoral and tibial tunnels, providing sufficient mechanical strength to maintain knee stability.^8^ However, it has a relatively high incidence of complications such as patellar fracture, patellofemoral chondromalacia, and anterior knee pain.^9^, ^10^ As an alternative to BPTB, the quadriceps tendon with a patellar bone block exhibits superior biomechanical properties.^11^ In the early postoperative period after ACLR, bone‐to‐bone healing on the femoral side meets the core requirement of no graft micromotion during knee rehabilitation, effectively reducing the risk of graft loosening, with satisfactory clinical outcomes.^12^ Nevertheless, this technique requires specialized instruments for precise preparation of the bone block and rectangular femoral tunnel, leading to higher surgical costs and technical demands. The most commonly used hamstring tendon graft involves a small harvest area and reduces the incidence of complications such as anterior knee pain and extension dysfunction.^13^, ^14^ However, tendon‐to‐bone healing occurs between the hamstring tendon and the femoral/tibial tunnels, which is inferior to bone‐to‐bone healing in terms of stability and healing rate, making it unsuitable for patients requiring early high‐intensity activity.^15^ From a biomechanical perspective,^16^, ^17^ the rate of rerupture at the femoral site after ACLR (particularly with hamstring tendon grafts) is approximately 42%. This may be attributed to the “windshield‐wiper effect” between the graft and the femoral tunnel aperture during knee flexion from 60° to 90°, with repeated friction leading to elevated local stress in the femoral tunnel.^18^ Based on biomechanical characteristics and the advantages and disadvantages of mainstream grafts, our team proposed using an ITB graft with a bone plug for ACLR. The bone block design further enhances graft stability within the femoral tunnel, effectively attenuating wear and micromotion caused by the windshield‐wiper effect. Furthermore, human subject research has revealed^19^, ^20^ that in standard anatomic single‐bundle ACLR, the overall graft strength at 6 months postoperatively is approximately 50% to 70% of that of the native ACL. The femoral tunnel attachment area, because of stress concentration, may exhibit an even lower strength (approximately 15‐20 MPa). Therefore, positioning the bone block at the femoral end promotes osseous healing within the tunnel and reduces the incidence of femoral‐side rerupture. The bone block‐tendon junction presents a short oblique bone surface, which can match the oblique contour of the femoral tunnel aperture, achieving a favorable interface fit (Figure 5). Tendon fixation on the tibial side avoids excessive tibial tunnel widening caused by bone block implantation and simplifies surgical steps while shortening the operative time. The fibrous tissue of the ITB exhibits good extensibility and tailorability;^21^ it can be woven into various diameters according to the diameter and length of the femoral and tibial tunnels. Particularly for patients with an enlarged tunnel diameter, the cross‐sectional area and initial mechanical strength of the graft can be increased by enlarging the bone block diameter and fold‐weaving the ITB. The ITB with a bone block is harvested from a nonweight‐bearing and noncore motor region. Partial fiber harvest has minimal impact on knee flexion‐extension and lower extremity muscle strength, thus avoiding complications such as anterior knee pain and knee flexion weakness.^14^ During dissection, graft harvest with bone block is performed under direct vision, with bone tissue only taken from the margin of Gerdy's tubercle (size ≤1 × 1 cm), avoiding injury to the core attachment area and effectively preventing postoperative lateral instability^22^, ^23^ (Table 4).

**FIGURE 5:**
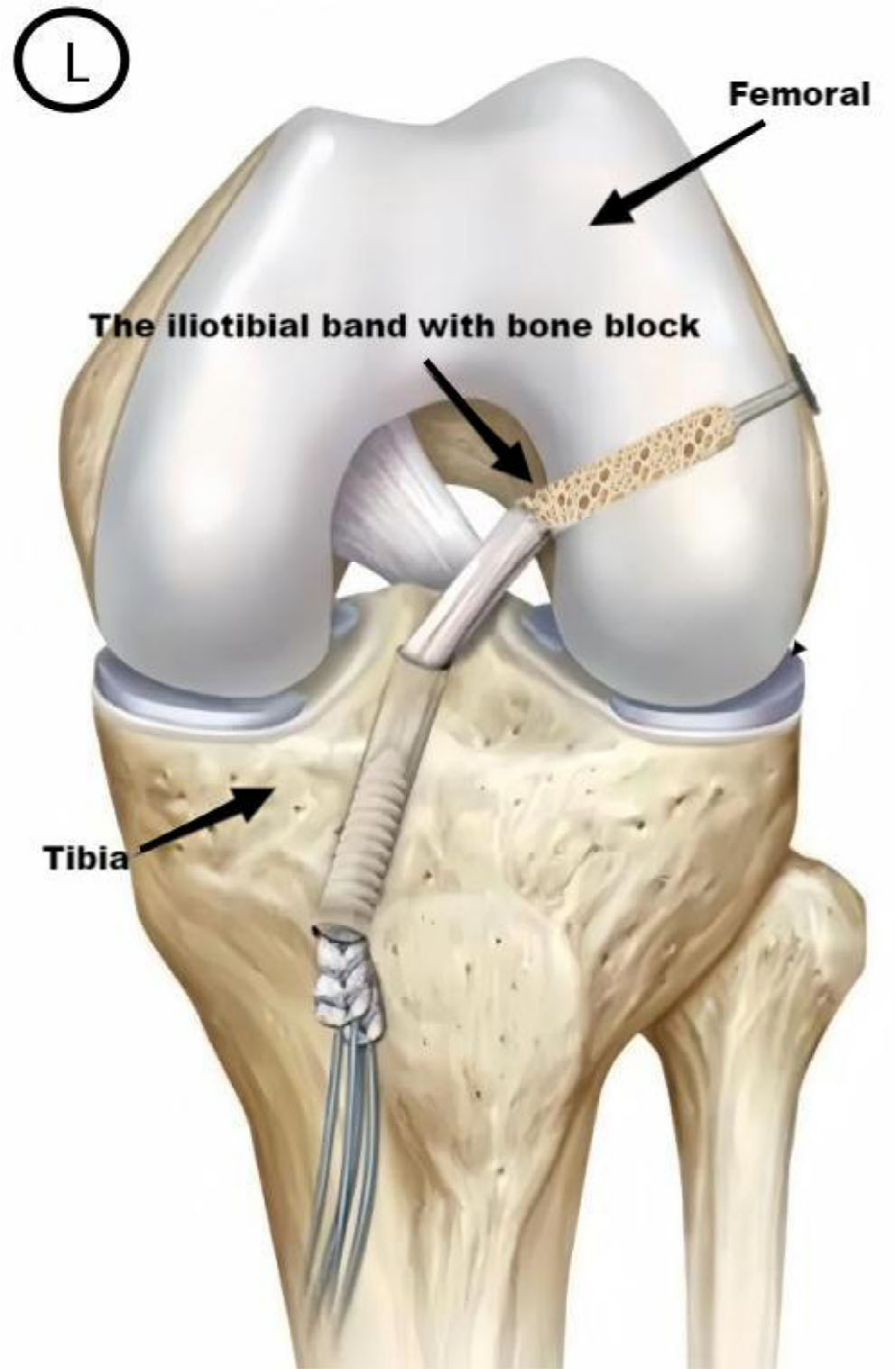
Schematic diagram of the completed graft reconstruction, showing the oblique anatomical matching between the bone block and the femoral tunnel aperture. (L, left.)

**TABLE 4:** Advantages and Disadvantages

Advantages
• The bone‐to‐bone healing at the femoral side features rapid healing and high mechanical strength, which minimizes graft micromotion, reduces the risk of rerupture at the femoral end, and alleviates femoral tunnel abrasion and tunnel widening.
• The surgical procedure is technically straightforward with a relatively gentle learning curve.
• It avoids common donor‐site complications of BPTB grafts, including anterior knee pain, pain during kneeling, and patellar tendinitis.
• It eliminates the postoperative deficits associated with hamstring tendon grafts, such as knee flexion weakness and compensatory quadriceps atrophy.

Disadvantages
• The bone block is prone to splitting during insertion into the femoral tunnel, which may prevent full seating of the graft.
• There is a potential risk of anterolateral complex injury, which requires strict control of bone harvesting range.

BPTB, bone–patellar tendon–bone.

Therefore, the use of the ITB graft with a bone block for ACLR is characterized by simple manipulation, convenient tendon harvesting, and favorable biomechanical properties. Osseous healing on the femoral side provides high mechanical strength, and this design allows for faster healing and higher initial stability, which is conducive to early rehabilitation. Moreover, in revision cases with pre‐existing enlarged bone tunnels, the bone block can achieve firm press‐fit fixation within the bone tunnel and effectively fill bone defects. It also provides a graft option for patients requiring revision after primary or multiple failed ACLRs.

## DISCLOSURES

The authors (D.H., W.W., J.W., Z.Y., G.W., X.C., J.H.) declare that they have no known competing financial interests or personal relationships that could have appeared to influence the work reported in this paper.
